# Phytochemicals: “A Small Defensive Advantage for Plants and Fungi; A Great Remedy for the Health of Mankind”

**DOI:** 10.3390/molecules26206159

**Published:** 2021-10-12

**Authors:** José A. Lupiáñez, Eva E. Rufino-Palomares

**Affiliations:** Department of Biochemistry and Molecular Biology I, Faculty of Sciences, University of Granada, Avenida Fuentenueva, 1, 18071 Granada, Spain

In the chronology of Biochemistry, as a new science that emerged in the mid-nineteenth century after its separation from Organic Chemistry and Physiology, its beginnings were characterized by an intense search and subsequent isolation and characterization of different organic compounds that were part of the chemical composition of living organisms. Scientists, such as Schwann, Pasteur, Berthelot, Bernard, Liebig, Wöhler and Büchner, played a fundamental role in these origins. For example, Schwann discovered in 1836 that gastric fluid contained, in addition to hydrochloric acid, another digestive component that he called pepsin, or Wöhler, who obtained in 1828 an organic molecule, urea, using the method of chemical synthesis that bears his name. All these initial studies formed what, for a long time, was known as Elemental and Structural Biochemistry. The compilation of all these organic elements for more than a hundred years served as the chemical basis for the classification of most of the natural compounds present in the biosphere [1].

The next step in the “construction” of Biochemistry, well into the 20th century, was an intensive study of the functionality and dynamics of each of the organic molecules present in living beings. Some of them are used as ashlars in the construction of macromolecules destined, mainly, in the formation of the macrostructures of the living being, while others play an effective functional and dynamic role. In this way, the circle of the structure-function binomial, basic for the understanding of all biological sciences, is closed. All this constitutes what is known as Functional and Dynamic Biochemistry, and although the first known vestiges of metabolism date back to the middle of the 13th century by the hand of a doctor from Damascus called Ibn al-Nafis (1213–1288), who stated, in his best known work, *Theologus Autodidactus*, that “... the body and all its parts are in a continuous state of dissolution and nutrition, so they are inevitably in permanent change”, its greatest exponent was HA Krebs, Nobel Prize in Physiology or Medicine in 1953, after the publication in 1932 of his work on the discovery of the urea cycle, a cyclical metabolic process through which different nitrogenous compounds, mainly amino acids, are processed when they are not recycled, generating urea as a final product [2].

From a functional point of view, a good example of the latter class of molecules is a group of natural compounds known as “phytochemicals.” An extraordinary, and almost inexhaustible source of these compounds is made up of a large part of terrestrial and aquatic plants, along with numerous species from the kingdom of fungi. Over time, it has become known that these molecules play a decisive role in the defence of these plants and fungi against herbivores, competitors, pathogens and predators, preventing, on the one hand, recurrent infections caused by parasites, bacteria and viruses, and on the other, of the destruction of the organism produced by different types of phytophages and mycophages [3]. A large number of these compounds are found in foods and although they are not considered as nutrients or macronutrients, nor are they included in the group of vitamins or minerals, they provide various beneficial functions. That is why foods that contain them are called functional foods since, in addition to their nutritional component, they also provide other types of health benefits.

In view of how the development of phytochemicals has evolved, and paraphrasing the recapitulation theory of Ernst Haeckel, who in 1866 defended that “…embryonic ontogeny is a brief and rapid recapitulation of phylogeny”, it can be said, in a certain way, that the history of phytochemicals recapitulates the history of biochemistry; first it was the discovery of molecules together with the decipherment of their structure and later, the recognition of their functions and bioactivities. At present, and especially in the last 20 years, there is a real explosion in the search for these compounds, capable of presenting important and abundant biological properties. Although many of them act as real poisons and teratogens, many more are being investigated thanks to their beneficial effects on health and can be used as new drugs or adjuvants in the treatment of various diseases [4]. Taking advantage of the anti-inflammatory capacities of many of them, their therapeutic use could be very useful in all those infectious diseases with a huge increase in cytokines, as is the case of the pandemic produced by SARS-CoV-2. In this sense, in several countries a clinical trial is being carried out using maslinic acid (pentacyclic triterpene) together with hydroxytyrosol (polyphenol), which have both demonstrated high antiviral and anti-inflammatory capabilities, to treat COVID-19 patients.

The classification of organic compounds has always been very ambiguous and that is also the case of phytochemicals. However, five groups can be included among them as the most significant: flavonoids, phytosterols, terpenoids, lignans and stilbenes [5], all of which are recognized for their large number of bioactive effects as nutraceuticals, essential nutrients and even allelopathic, thus influencing both the growth and survival, as well as the reproduction of other organisms [6]. Among all these phytochemicals, polyphenols and triterpenoids should be highlighted, as many of their bioactive capacities are known, both in vitro and in vivo [4]. Many of these compounds have various beneficial properties, such as anticancer, antiproliferative and antiangiogenic in different tumour lines [7,8,9,10,11,12,13,14,15], as well as antioxidant and anti-inflammatory [16,17,18,19], while others have properties related to metabolic syndromes, such as antidiabetogenic and cardio and neuroprotective [20,21,22], others anti-infectious, such as antifungal, antimicrobial, antiviral and antiparasitic [23,24,25], and, finally, others that affect organic and metabolic activity, such as growth inducers, activators and inhibitors of enzymatic activity [26,27,28,29,30], as well as modulators in the production of reducing equivalents in the form of NADPH. This explains most biosynthetic, cellular and organic growth, nutritional processes, as well as processes of differentiation, cellular detoxification and oxygen free radical scavenging [31,32,33,34,35,36,37].

On the other hand, the process of organic synthesis has been used recently with increasing intensity, with the objective of finding chemical derivatives of natural compounds that present improvements in functional effectiveness in relation to the bioactivity of the original compound. In this sense, different chemical groups are being used in the synthesis of these derivatives, significantly increasing their effectiveness [4]. Among these groups, acyls, aminoacyls and dipeptidyls [23,24,38,39], derivatives of pegylated and diamino-pegylated groups [40,41,42,43,44], and even derivatives with coumarin [45], with which their activities are significantly increased, stand out.

After a rigorous peer review process, the Special Issue entitled Natural Products and Derivatives in Human Disorders, (https://www.mdpi.com/journal/molecules/special_issues/natural_products_chemoprevention, date accessed 20 January 2019), compiles a total of seven original articles in which different phytochemicals and chemical derivatives are used, delving into the study of the molecular roles they present in different human pathologies, such as cancer, neuronal and ocular dysfunctions, arterial hypertension and osteoarthritis. A summary of the main characteristics of the original papers included in this Special Issue, both in terms of provenance and molecular type, as well as their molecular effects, are discussed below.

It is relatively frequent that pathologies related to the production of tears by the lacrimal glands can occur both in elderly people and in patients with certain types of autoimmune diseases or even by external aggressions such as chemical or thermal burns. When this problem worsens, the well-known dry eye disease (DED) or dry keratoconjunctivitis appears, the main pathology of which consists of corneal ulcers and infections. The most common treatment is to moisten the eye with artificial tears composed basically with hyaluronic acid, or with lubricating ointments; Kang et al. [46], in a very interesting study, shows the beneficial effects of the extract of *Aucuba japonica* or spotted laurel, together with its bioactive compound, aucubin, an iridoid glycoside, that is, a monoterpene glycosylated derivative. In their study, these authors demonstrate that in in vitro assays, human corneal cells (PCS-700-010), exposed to desiccation stress and treated with these compounds, extract and active ingredient, increase their survival in a clearly dose-dependent effect. At the same time, overexpression of mRNA levels, generated by ocular desiccation, of different inflammatory cytokines (interleukins 1β (IL-1β) and 8 (IL-8), and tumor necrosis factor TNF-α) were significantly reduced by the administration of the extract and aucubin. Moreover, in in vivo tests using SD (Sprague-Dawley) rats as an animal model, they found that after unilateral excision of the exorbital lacrimal gland, both lacrimal volumes and corneal irregularities recovered completely. In addition, they found that these compounds significantly reduced apoptotic cells in the cornea. All these results strongly suggest that these compounds can be considered as a novel therapy for this disease and that aucubin is probably responsible for this effect.

The evolutionary appearance of vertebrate animals generated the need to implement an efficient bone turnover system that allows them to carry out an adequate regeneration of the entire osteoarticular system. To this end, it is essential to ensure a regulated and constant synthesis of collagen. A key element for this is a constant and adequate supply of glycine molecules [47,48]. However, the cellular synthesis of this amino acid is greatly reduced as it is linked to the concomitant synthesis of coenzymatic forms of tetrahydrofolic acid (THF), preventing the production of sufficient and adequate amounts of this amino acid, thus fulfilling the restriction theorem of glycine biosynthesis which states: “*If the only significant metabolic pathway for glycine synthesis is the reaction catalyzed by glycine hydroxymethyl transferase, the steady-state metabolic flux for net glycine production cannot, under any condition, exceed the flux for the consumption of the C1 units transferred* via *N^5^-N^10^ methylene THF*”. Glycine must therefore be considered an essential amino acid because the capacity for its synthesis is much lower than its actual requirement and, as a consequence, the inevitable onset of degenerative diseases such as osteoarthrosis, osteoporosis and osteoarthritis [47,48]. Therefore, all these bone diseases are inevitably linked to the growth and development of large vertebrates. Within metabolic theory, this evolutionary “failure”, especially frequent and serious in large vertebrates, is classified as a so-called “default metabolic error”. In this context, traditional Korean medicine has been using an herbal preparation known as RyuPungHwan (RPH) consisting of extracts of seven different plants, *Astragalus membranaceus, Turnera diffusa, Achyranthes bidentata, Angelica gigas, Eclipta prostrata, Eucommia ulmoides* and *Ilex araguariensis*, with high content of flavonoids and triterpenoids, in patients with osteoarthritis, not to cure the disease but to alleviate the inflammatory process and pain. Taking into account this background, Hong et al. [49], have studied the effects of this herbal preparation in SW1353 chondrosarcoma cell models, taking into account the inflammatory process associated with the disease. Administration of this preparation produced a significant inhibition of Interleukin-1β-stimulated inflammation, making two active compounds isolated from the herbal preparation, isomucronulatol 7-O-β-D-glucoside, a flavonoid derivative, and ecliptasaponin A, a triterpenoid derivative, responsible for this clear anti-inflammatory effect. They showed that both the preparation and the two identified compounds were able to reduce the expression of matrix metalloproteinase 13 (MMP13), cyclooxygenases 1 and 2 (COX-1 and -2), Tumor necrosis factor α (TNF-α), Interleukin-1β (IL-1β) or protein p65, previously increased by the administration of IL-1β to SW1353 cells.

Hypertension, which is very common nowadays, is a serious risk factor associated with cardiovascular diseases, which, together with other diseases such as obesity, diabetes and atherosclerosis, constitute the characteristic tetrad of the metabolic syndrome. In this context, Hong et al. [50], belonging to the same research group as the previous work, analyze the effects on the main molecular markers of hypertension of a plant preparation, commonly used in traditional Korean medicine and consisting of three species, *Pine densiflora, Annona muricate* and *Momordica charantia*, called No-ap (NA), as well as several of its main bioactive molecules, a roseoside (a compound belonging to the group of C13-norisoprenoids generated by the degradation of 40-carbon terpenes) and the flavonoid, icariside E4. The authors used as a biological model H9C2 cells, rat cardiomyocytes derived from myoblasts, treated with angiotensin II as a hypertensive molecule; with an antihypertensive drug, termisartan; with gingenoside as a positive control; with different doses of the NA extract; and with the two isolated compounds (roseoside and icariside), both individually and together. Treatment with angiotensin II resulted in a significant increase in myocyte angiotensin II receptor 1, (AT1), tumor necrosis factor α, (TNF-α), monocyte chemoattractant protein 1, (MCP-1), tumor growth factor β, (TGF-β) mRNA expression levels, NADPH oxidase enzyme activity, H_2_O_2_ and superoxide ion (•O_2_^−^) levels, while the activity levels of the antioxidant enzymes catalase and superoxide dismutase were significantly reduced. Subsequent treatment with the extract and the isolated components restored the levels of all markers to control values. The effect found was dose-dependent and, in addition, a synergistic effect of the active compounds was observed. The authors conclude that hypertension therapy with these compounds should be considered for further clinical trials.

Neurodegenerative diseases constitute, nowadays, a serious problem, afflicting people of all ages, but especially older people, preventing them from adequate cognitive development and thus seriously affecting their life and daily activities. Many are classified as neurodegenerative diseases and among the most important and visible are Alzheimer’s disease and dementia with Lewy bodies, as well as Friedreich’s ataxia, Huntington’s disease and Parkinson’s disease. In the first two, but especially in Alzheimer’s disease, one of the most important biochemical dysfunctions is the appearance of very high activity of the neurotransmitter degradation systems, mainly acetylcholine. Therefore, the role of cholinesterases, especially acetylcholinesterase and butyrylcholinesterase, plays a central role in the development of this disease. It is therefore of interest to develop potential drugs, based on natural compounds, that are capable of slowing down the rapid elimination of these neurotransmitters. In this regard, Loesche et al. [51] have succeeded in synthesizing up to 40 types of carboxamides, molecules derived from ethylenediamine, from up to five different naturally occurring triterpenes, oleanolic, ursolic, maslinic, betulinic and platanic acids. These authors evaluated acetylcholinesterase and butyrylcholinesterase activities using the Ellman assay and both enzymes were derived, respectively, from *Electrophorus electricus* and equine serum. Among all the carboxamides tested, seven different compounds showed inhibition constant (Ki) values in the nanomolar to micromolar range. The inhibitions are of mixed type with competitive dominance, according to the molecular modelling studies performed. Therefore, the synthesis of molecules with these characteristics could be used in the therapy of this type of neurodegenerative disease.

*Teucrium mascatense* is an understory shrub, the genus of which is widely distributed over several continents. Its medicinal value has been known since ancient times and, therefore, it has been used in both traditional and modern medicine, thanks to the presence of its bioactive components, including tannins, glycosides, phenols, steroids and terpenoids. Its bioactivities include antibacterial, antipyretic, anti-inflammatory, antidiabetic, antioxidant, analgesic and antipyretic properties. In this case, Panicker et al. [52] have investigated the anticancer capacity of extracts from this plant, as well as that of one of its main components, triterpene IM60. In their studies they used four cell lines, one for cervical cancer, HeLa and three for breast cancer, MCF-7, MDA-MB-231 and MCF-10A. To certify the possible antiproliferative activity, they studied the effects of the extract and the isolated triterpene on some of the most important apoptotic markers, such as cytotoxicity at different times and concentrations; levels of caspases 3, 7, 8 and 9; levels of PARP (Poly ADP ribose polymerase); measurements of cell apoptosis by Annexin V/Propidium iodide assays in order to decipher the different types of apoptosis, early, late and necrosis, as well as the morphological changes associated with apoptosis. The results indicate a clear anticancer effect by significantly increasing the levels of all molecular markers together with an increase in the apoptosis of malignant cells. This study identifies the triterpene IM60 as a potential drug that can be successfully used in breast cancer therapy.

If it is important that certain compounds have the ability to cure diseases, some of them as serious as cancer, in which irreparable individual and collective problems are caused, the ability to prevent them is an incalculable advance, both from the personal point of view and social, by being able to avoid all its fatal consequences. We know that many of the phytochemicals present in nature have this property and we just need to be able to find them and find it. This is the case of the good work presented by Juan et al. [53]. In it, the chemopreventive capacity of maslinic acid, isolated from *Olea europaea*, is studied in vivo in an animal model that mimics sporadic human colorectal cancer. Male Sprague-Dawley rats were used in this test, to which for 49 days they were administered, orally, different doses of maslinic acid, 5, 10 or 25 mg kg of animal weight. Subsequently, and after a week of rest, the cancer was induced by means of three weekly injections of 20 mg/kg of the carcinogenic inducer, 1,2-dimethylhydrazine. Under these conditions, different preneoplastic markers, aberrant crypt foci (ACF) and mucin-depleted foci (MDF) were analyzed. Since the administration of the lowest dose of maslinic acid, 5 mg/kg, decreases of 15% were found in ACF and up to 27% in MDF, achieving these significant decreases with the 25 mg/kg dose, with those that the reductions of these biomarkers were 33% and 50%, respectively, in addition, these results were corroborated by their association with the concentrations of maslinic acid found in the colon of the animals. The results of this work clearly demonstrate the preventive role that this phytochemical has on colon cancer, which allows us to conclude that with an adequate addition of this triterpene in the diet, this chemopreventive activity could be achieved, so important as to eliminate the problems inherent of this disease.

The inclusion of bioinformatics studies in biological research is essential to advance our understanding of the molecular mechanisms involved in the functions being sought. At present, a very high percentage of molecular biology studies use this essential tool [10]. The work presented by Lv et al. [54] falls within this experimental concept, in which experimental studies are combined with computational studies. In their work, the authors use a saponin (glycosylated triterpene), saikosaponin D, from a preparation of Chinese origin known as Radix Bupleuri, used as a traditional medicine for over 2,000 years in China, Japan, Korea and other Asian countries, and consisting of the dried roots of two plant species, *Bupleurum chinense* and *Bupleurum scorzonerifolium*. It has previously been reported that saikosaponin D is able to inhibit cancer cell growth by inducing apoptosis and arresting the cell cycle in the G_1_ phase. The present work aims to further investigate the molecular anticancer mechanism of this saponin by combining both network pharmacology and metabolomics databases. Up to 35 targets were studied in the bioinformatics analysis, selecting neuropilin-1 (NRP-1) for further investigation based on the degree of molecular docking score, demonstrating that saikosaponin D combined with NRP-1 deletion could significantly ameliorate HepG2 damage. As a consequence, metabolomics analyses showed that NRP-1 blockade exhibited the lowest metabolite deregulation score, and among the metabolites analyzed, carnitines and short- or long-chain phospholipids were mainly implicated. These results clearly implicate NRP-1 as a key molecule to explain the anti-hepatoma activity of saikosaponin D.

An example of the importance the use of different phytochemicals has had in recent years in order to help prevent, alleviate or, in its best version, cure the numerous pathologies that currently afflict humanity, is precisely the content of this Special Issue that we are commenting on. Diseases such as dry keratoconjunctivitis, osteoarthrosis, hypertension as a risk factor in cardiovascular diseases, the role of certain cholinesterase inhibitors in some neurodegenerative diseases, or some types of cancers, such as breast, colon and liver, are the subject of the works presented here. For all these reasons, the academic editors of this issue would like to sincerely thank all our colleagues, authors and reviewers, whose efforts and collaboration have contributed to the realization of this Special Issue, allowing it to become a reality.

## Data Availability

Not applicable.
